# What Matters in Cancer Survivorship Research? A Suite of Stakeholder-Relevant Outcomes

**DOI:** 10.3390/curroncol28040277

**Published:** 2021-08-20

**Authors:** Robin Urquhart, Sarah Murnaghan, Cynthia Kendell, Jonathan Sussman, Geoffrey A. Porter, Doris Howell, Eva Grunfeld

**Affiliations:** 1 Department of Community Health and Epidemiology, Dalhousie University, Halifax, NS B3H 1V7, Canada; sr438725@dal.ca (S.M.); geoff.porter@dal.ca (G.A.P.); 2 Department of Surgery, Nova Scotia Health, Halifax, NS B3H 2Y9, Canada; cynthia.kendell@ccns.nshealth.ca; 3 Department of Oncology, McMaster University, Hamilton, ON LV8 5C2, Canada; sussman@HHSC.CA; 4 Department of Supportive Care, Princess Margaret Cancer Centre, Toronto, ON M5G 2M9, Canada; doris.howell@uhn.ca; 5 Ontario Institute for Cancer Research, Toronto, ON M5G 0A3, Canada; eva.grunfeld@utoronto.ca; 6 Department of Family and Community Medicine, University of Toronto, Toronto, ON M5G 1V7, Canada

**Keywords:** cancer, survivorship, needs, outcomes

## Abstract

The outcomes assessed in cancer survivorship research do not always match the outcomes that survivors and health system stakeholders identify as most important in the post-treatment follow-up period. This study sought to identify stakeholder-relevant outcomes pertinent to post-treatment follow-up care interventions. We conducted a descriptive qualitative study using semi-structured telephone interviews with stakeholders (survivors, family/friend caregivers, oncology providers, primary care providers, and cancer system decision-/policy-makers) across Canada. Data analysis involved coding, grouping, detailing, and comparing the data by using the techniques commonly employed in descriptive qualitative research. Forty-four participants took part in this study: 11 survivors, seven family/friend caregivers, 18 health care providers, and eight decision-makers. Thirteen stakeholder-relevant outcomes were identified across participants and categorized into five outcome domains: psychosocial, physical, economic, informational, and patterns and quality of care. In the psychosocial domain, one’s reintegration after cancer treatment was described by all stakeholder groups as one of the most important challenges faced by survivors and identified as a priority outcome to address in future research. The outcomes identified in this study provide a succinct suite of stakeholder-relevant outcomes, common across cancer types and populations, that should be used in future research on cancer survivorship care.

## 1. Introduction

Nearly two-thirds of all individuals diagnosed with cancer today will survive long-term [1]. The rising number of cancer survivors is due to both increasing cancer incidence as well as improved survival through earlier detection and better treatments. Despite enhanced survival, cancer and its treatment have substantial late and long-term adverse impacts. After treatment, the magnitude of the survivors’ medical and supportive care needs is similar to the magnitude of needs they experience during treatment [2]. As a result, follow-up after cancer treatment is a recognized component of medical care. In a seminal 2006 report, the Institute of Medicine (IOM) in the United States outlined essential components of follow-up care: prevention and detection of recurrence and new cancers; prevention and management of the impacts of cancer and its treatment; and coordination amongst specialty and primary care to ensure that all of the survivor’s health needs are met [3].

Since the IOM report, many interventions have been tested, in both research and practice settings, to improve the survivors’ outcomes during follow-up care. However, the reality is that many studies lead to null or mixed results [4,5,6], and survivors continue to lack access to timely information and support during the follow-up period, have high anxiety and fear after treatment, experience poor coordination across health sectors, and feel unprepared for follow-up including the physical, psychological, and practical effects of cancer and its treatment [7,8,9,10,11]. Importantly, studies of follow-up care interventions have typically assessed clinical endpoints, patient/provider satisfaction, and quality of life. However, such outcomes may not address the outcomes most important to survivors themselves and other important stakeholders. For example, two recent studies revealed a mismatch between the outcomes assessed in RCTs of survivorship care plans and the outcomes stakeholders identified as important [12,13]. Others have found that outcomes important to patients are overlooked or viewed as unimportant by researchers [14,15]. A mismatch between researchers’ expectations of a particular intervention versus stakeholders’ expectations may result in the selection of inappropriate study outcomes and therefore null findings. This study sought to identify stakeholder-relevant outcomes pertinent to follow-up care interventions. Stakeholders included survivors, family/friend caregivers, health care providers, and health system decision-makers. The intent was to identify a suite of stakeholder-relevant outcomes to be incorporated in future follow-up care studies.

## 2. Materials and Methods

We conducted a descriptive qualitative study [16] using semi-structured telephone interviews with stakeholders across Canada. The qualitative description was concerned with summarizing and describing the informational contents of the data. Data were grounded in the perspectives of participants with minimal interpretation. Stakeholder groups included survivors, family/friend caregivers, oncology providers, primary care providers, and cancer system decision-/policy-makers. Participants were purposively sampled to achieve maximal variation in cancer type, place of work or residence (urban/rural/remote), and special populations (e.g., pediatric, adolescent, and young adults).

Oncology providers, primary care providers, and decision-/policy-makers were recruited via cancer programs/clinics across Canada; provincial and national organizations, groups, and networks (e.g., Canadian Partnership against Cancer, provincial cancer agencies); and publicly available information in each province and territory (e.g., leads of primary care and oncology networks). Participants were also asked to identify individuals within their organizations who had an interest in cancer survivorship/follow-up care using a non-probability snowball sampling technique. The PI [RU] initially approached all potential participants via email. If the participant responded in the affirmative, the research associate [SM] followed up to discuss the nature and purpose of the study and to arrange a time to conduct the informed consent discussion and interview. If the potential participant failed to respond to the initial contact within one week, a second follow-up email was sent.

Survivors and family/friend caregivers were recruited by distributing study information through cancer, cancer survivor, and caregiver organizations. This included support groups and networks, and patient advocacy organizations. Interested persons were instructed to contact the research associate by telephone or email to acquire further information about the study. If the person wished to participate, the associate arranged a time to conduct the informed consent discussion and interview.

Data were collected by the research associate via one-on-one, semi-structured, telephone interviews. The interviewer had no prior relationship with any participants. The interview guide (Supplementary File S1) was developed based on the study objectives using practical guidance from Patton [17] and Rubin and Rubin [18]. Participants were asked to reflect on what is most important in the post-primary treatment phase of care and what interventions would ideally improve for survivors, family/friend caregivers, providers, and health systems. Recruitment continued until data saturation was achieved. Each interview was audio-taped to ensure the data were captured in true form, and transcribed verbatim by an experienced transcriptionist. No repeat interviews were conducted.

Data analysis involved coding, grouping, detailing, and comparing the data by using the techniques commonly employed in descriptive qualitative research [16]. In general, these techniques involved the coding of data; recording insights and reflections on the data; sorting the data to identify similar patterns and important features; identifying commonalities and differences across the dataset and extracting them for further analysis; iteratively determining a concise number of categories that hold true for the data; and examining these in light of existing knowledge. To begin this work, two researchers [RU, SM] independently reviewed and coded three transcripts to identify salient concepts. They then developed a codebook consisting of both deductive, guided by the National Cancer Institute Office of Cancer Survivorship topics in cancer survivorship [19], and inductive concepts. The research associate [SM] used this codebook to code several additional transcripts, allowing for the expansion and merging of codes as necessary. Upon agreement of codes, the research associate then coded the remaining transcripts with regular meetings with RU to review and question the coding. Outcome categories were sorted and compared across stakeholder groups to identify commonalities across groups as well as across types of cancer, as the aim was to identify a suite of stakeholder-relevant outcomes applicable across (at least most) cancer types. The analysis was performed manually with the assistance of qualitative software (NVivo) for data management and to facilitate comparison and synthesis of codes.

## 3. Results

Forty-four participants took part in this study. Table 1 presents the participant demographics. Just over 40% were cancer survivors or family/friend caregivers, whereas the remaining were health care providers or decision-makers. Participants represented the Western, Central, and Atlantic provinces. Of the survivors/caregivers, most were female (72.2%) and were three or more years post diagnosis (66.7%). Four health care providers had a specific interest or expertise in pediatric, adolescent, and young adult cancers, while two survivors and one family/friend caregiver had lived experience with a young adult cancer. Stakeholder relevant outcomes fell into five outcome categories: psychosocial, physical, economic, informational, and patterns and quality of care. Table 2 presents the 13 stakeholder-relevant outcomes in summary form.

### 3.1. Psychosocial Outcomes

Participants repeatedly described three psychosocial outcomes that were particularly relevant to follow-up care: reintegration after cancer treatment, fear of cancer recurrence, and anxiety. One’s reintegration after cancer treatment was described by all stakeholder groups as one of the most important challenges faced by survivors and therefore identified as a priority outcome to address in future research. This reintegration was described as adjusting to a new normal, and characterized by ongoing challenges associated with changes to physical functioning or appearance, emotional well-being, and social relationships and roles. For example, related to relationships, one participant said:

A lot of people aren’t prepared for the culture shock. That they may have had a great relationship with somebody for, you know, 5, 10, 20 years, and all of a sudden that relationship doesn’t exist. Or even partnerships, you know, marriages…

[P4 Survivor]

Related to adjustment, survivors also talked about the difficulty of feeling comfortable in, and trusting, one’s body again after a cancer diagnosis:

It’s still your body that has betrayed you. And you get this very weird sensation where you’re walking around going, ‘I don’t trust me anymore.’ You just become disembodied. Your body is now the enemy. And it’s a very, very strange place to be in your head, to be thinking this body—you know, it’s the only body I have—is now something I don’t trust. That’s hard. That’s a real hard one.

[P7 Survivor]

Finally, many participants recognized the challenges that survivors face returning to life after treatment when those around them expect them to easily resume their pre-cancer roles and activities once treatment has finished:

I think they struggle to say, ‘okay, I’m over my acute treatment, how do I sort of get my life back on track, how do I become normal?’ Because there’s expectations from those around them that suddenly they’re done and they can assume those roles.

[P22 Health care provider]

Fear of cancer recurrence was emphasized by nearly all participants as being especially prevalent after treatment. As participants stated, “everybody’s worried about recurrence” [P13 Health care provider], “the fear of recurrence is huge” [P2 Decision-maker], and “I just kind of feel like we’re waiting for it to come back” [P29 Caregiver]. Participants discussed greater fear in the time period approaching follow-up tests and investigations, and subsequently awaiting results. As one participant explained:

The stress of, the anxiety of what normally occurs in the two weeks or so before your appointment, you know, when you’re wondering what the results are going to be, was probably doing more harm to me than anything that they could help me with in terms of the visits. You dream of the day when you’re no longer getting those tests because they are very stressful. You know, the week or so before the test, like you’re so hyper aware of every single symptom.

[P7 Survivor]

Participants also discussed how age can influence one’s level of fear of recurrence, with older adults sometimes less impacted by the fear even when it is present. As one participant said:

The only emotional thing, I guess, is, you know, in the back your brain is ‘has the cancer been wiped out because of the chemo or is it going to come back? When is it going to come back, if it comes back?’ But that’s tempered by the fact that I’m 75 years old. And if anything, this process has done is it’s made me come to grips with the fact that life is a certain period of time and it will end. I guess the conflict in your brain is when and how?

[P24 Survivor]

Finally, all participants discussed the need to address the high anxiety that most survivors experience in the post-treatment period. Anxiety was commonly discussed in relation to a number of activities and events that occurred after completion of primary treatment. The first was the sudden reduction in visits that occurred once treatment has ended, leading many survivors to feel that their “safety net” had been taken from them and that their care might “fall through the cracks.” One participant put it this way:

You know, take a breast cancer patient, for example. Radiation could be at the end of their treatment. And so they’re coming every day for say a number of weeks. And then all of a sudden treatment ends. And so they often will report losing that sort of safety net that they had.

[P33 Decision-maker]

The second event that participants discussed as triggering anxiety was discharge from the cancer center back to community-based care. As one participant put it, “There’s a lot of fear. They feel that they’re very well cared for when in the clinic, in the cancer system, but then once they’re discharged, they feel left to themselves. And it’s quite frightening for them”.

[P2 Decision-maker]

### 3.2. Physical Outcomes

Despite a wide range of physical needs depending on cancer type and treatment, three physical outcomes were described across stakeholder groups as particularly important to address: fatigue, cognitive impairment (“chemo brain” or “brain fog”), and sexual health. Both fatigue and cognitive impairment were commonly discussed across all stakeholder groups as being highly prevalent and challenging for survivors to manage after treatment. These ongoing impacts were viewed as important physical concerns that impede survivors’ abilities to reintegrate into life after treatment, particularly returning to work. One participant said, “I would say fatigue is usually always one of the biggest complaints from patients … a lot of it is an emotional and physical sort of fatigue” [P10 Health care provider]. Another discussed her difficulties with cognitive impairment:

I referred myself to a psychologist because I had…I really couldn’t read and I couldn’t do more than one thing at a time. And she allowed that sometimes it’s a very short period, sometimes it’s a lifetime of having that kind of confusion—foggy brain or I don’t know what they call it. … It took me years to get my speed up. I learned that, no, I couldn’t do more than one thing at a time. I think we’ve counted seven times I burned carrots because I wasn’t…I was doing something else while I was cooking supper.

[P12 Survivor]

Sexual health was commonly described as an after effect of a cancer diagnosis or treatment that is prevalent and requires considerable attention. Sexual health concerns ranged from sexual dysfunction to fertility challenges to changes in sexual intimacy and relationships. Participants discussed the latter concerns (fertility, changes in sexual intimacy/relationships) as being particularly prevalent in younger cancer survivors. As one participant discussed:

If they haven’t already been thinking about or talking about sexuality and intimacy, then as they move into that beyond treatment phase, trying to kind of get their feet under them again and figure out their new normal, that starts to surface more so. And then I hear more about, you know, how do I, in my relationship, move to a different level? I’m, you know, still sore. My husband is ready to move on. I’m not there yet. How do I manage that? Or, because I’ve worked with people dealing with breast cancer, and certainly some of them had a significant body image change, you know, we’ll talk about some of the challenge of getting comfortable with their own body again and comfortable enough that they’re comfortable with their partner seeing them that way. … and for some, the whole dating scene can be a challenge.

[P45 Health care provider]

### 3.3. Economic Outcomes

Participants discussed two outcomes of particular importance in terms of practical and economic sequalae after treatment: return to work and financial burden. Regarding the former, many participants discussed how physical challenges such as fatigue and cognitive impairment make it especially difficult for survivors to return to work. As one caregiver noted:

Her return to work was like, a little bit difficult because she has this chemo brain or whatever that they refer to it. And she finds that frustrating at times because simple things that you just take for granted after doing them a hundred times, all of a sudden like you can’t remember it or remember how to do it. It’s just, like, it’s gone.

[P23 Caregiver]

They also discussed how return to work is intertwined with returning to life after treatment, and connected to both a return to normalcy as well as financial stability. As stated by one participant:

There’s the new normal concerns. What’s my life going to be like? When am I going to get to do my normal activities? Will I be able to go back to work? And, of course, there’s the socioeconomic ones around if I can’t go back to work, how am I going to have money to pay my bills?

[P3 Decision-maker]

Participants frequently discussed financial burden due to a cancer diagnosis. This was sometimes linked to concerns about employment. It was also discussed in relation to rural/urban differences in access to care whereby patients in rural areas often incur more out-of-pocket costs for cancer treatment as well as needed support after treatment. One participant said:

Some of these people, especially up north, are travelling for days to get to an appointment, and staying overnight in a hotel. And, you know, there’s usually a family or caregiver with them. You know, that’s a lot of time and money and energy for maybe a 10 minute appointment to hear that you’re doing great.

[P14 Decision-maker]

Many survivors and caregivers noted that these economic concerns were lower if the patient was older and retired at the time of their cancer diagnosis. As one survivor said, “So the cancer never held me back in terms of career and money. Financially, it was never a burden, which I know is for somebody caught in the middle of their career” [P12 Survivor].

### 3.4. Informational Outcomes

Participants described two outcomes they deemed relevant with respect to information provision: knowing what to expect and informational continuity. All participants discussed the importance of knowing what to expect in the survivorship period to optimize health and overall well-being. This included knowing one’s surveillance schedule, knowing what signs and symptoms may indicate recurrence, knowing what long-term and late effects might happen and how to mitigate these, knowing about available resources and services, and knowing that life after cancer may not return to a pre-cancer normal. As stated by one participant, “I think the biggest issue for patients is really a lot of that uncertainty of what to expect. When are things going to go back to normal for them?” [P33 Decision-maker]. Knowing what to expect was also a particular concern of caregivers, who want this information to best support their loved one:

She would leave the room and I would go in and ask kind of like my questions. I felt like the communication kind of broke down after [treatment]. So there was really a lack of information for me as a caregiver as kind of like what are the next steps as far as like, you know, your cancer treatments are done.

[P29 Caregiver]

Informational continuity and ensuring consistent, clear information across all members of the care team (specialists, primary care providers, and survivors/families) was also seen as a critical outcome in survivorship care. As stated by one participant:

I think it boils down to making sure that everyone’s on the same page. So patients, primary care providers, and the cancer specialists are on the same page in terms of what to look out for, what follow-up needs to be done, what tests need to be done, who’s in charge? And I think that’s really the most important piece of it … I think the most important piece is that information gets relayed to the right people.

[P15 Health care provider]

### 3.5. Patterns and Quality of Care Outcomes

Participants described many system issues and discussed three outcomes in particular that they deemed relevant with respect to survivorship: access to care, coordination of care, and use of existing clinical practice guidelines/evidence-based practices, supports, and services. Participants discussed substantial differences in access to rehabilitation and supportive care specialists and services based on where a person resides (i.e., urban versus rural differences). One participant described their barriers with access to care, “And with the dynamics of Saskatchewan … I mean we have a lot of barriers that people from up north face with language and travel for treatment. So, I mean I traveled two and a half hours [to receive care]” [P4 Survivor].

Related to access to care, participants also noted differences in one’s ability to access needed services and support based on one’s ability to pay out-of-pocket. Many survivors and families do not have financial means to access services that they require during survivorship because these services exist outside of hospital settings. One participant said, “Like I guess…I don’t know if it’s just the way it’s run or just lack of finances. But you know, there’s a lot of things I think you have to look outside the actual hospital or the clinic for” [P23 Caregiver]. Others discussed a reduced ability to access services such as physiotherapy, prosthetic care, sexual health supports, and other supportive care services due to financial constraints.

Participants also discussed coordination of care—both coordination of information as well as responsibility—and the importance of designing and implementing interventions that improve coordination across oncology and primary care. Coordination of care was seen as particularly germane to people with more complex care issues such as older adults with multimorbidity, who see multiple specialists for their health care. Many family/friend caregivers expressed their experience, for example, that there was a lack of information transfer and role clarity across their loved one’s care providers. As one participant described, “We do a lot of sharing information ourselves, and reports and that, with our family physician as opposed to it coming directly from the specialist” [P44 Caregiver]. Another said:

There was a lapse between that and kind of follow-up or regular appointments with her GP. So, she was tapped in with her oncologist but didn’t see her GP for probably two years. And it turned out that she had some kind of heart condition that slipped through the cracks.

[P42 Caregiver]

Both decision-maker and health care provider participants emphasized the need to investigate the use of evidence-based practices, support, and services. This stemmed from the fact that they were keenly aware of, and described, wide variation in follow-up care practices as well as in access to services and supports (e.g., physical activity programs, psychological counselling) needed during follow-up care. While they recognized that guidelines for follow-up care exist, most discussed a lack of adherence to guideline recommendations across settings. In fact, many participants discussed the need to monitor practices as a way of improving the quality of follow-up care provided:

We have best care, best practices, in terms of models of care. So, they’re a broad set of recommendations. And we ask the regions to do a self-assessment…against those recommendations to see how they’re doing with respect to different aspects of providing follow-up care. And then at the beginning of the year and at the end of the year, what they did. And based on that, that’s kind of how we incentivize moving towards best practice for the cancer programs.

[P14 Decision-maker]

## 4. Discussion

This study aimed to identify a suite of stakeholder-relevant outcomes to be incorporated in future follow-up care studies. To do so, various stakeholders across Canada were asked to identify the outcomes they felt were most important in the follow-up care period. Thirteen outcomes were identified across stakeholder groups as being particularly important; these intersect across multiple care domains and at the system level. Several outcomes such as sexual health, financial burden, access to care, and coordination of care were perceived as being particularly relevant to some subgroups more than others. Together, this suite provides a short list of outcomes that should be studied in future research on cancer survivorship care.

Cancer survivors experience a wide range of physical, emotional, and economic needs after cancer treatment. A national, population-based survey study in Canada demonstrated that nearly nine in 10 cancer survivors had ongoing physical needs, nearly eight in 10 had ongoing emotional needs, and four in 10 had ongoing practical needs in the 1–3 year period after completing cancer treatment [20]. The most prevalent physical needs were fatigue, changes in sexual function, and changes in memory/concentration; the most prevalent emotional needs were anxiety and worry about cancer recurrence, followed by changes in sexual intimacy and depression; and the most prevalent practical/economic needs were returning to work/school, getting to/from appointments, and paying for health care. Thus, the findings from our qualitative study largely align with the most prevalent needs of cancer survivors in Canada [7,8,9,10,20] and elsewhere [21,22], and provide targetable outcomes in future research. Indeed, with the exception of sexual health, the psychosocial and physical outcomes identified in this study were also prioritized by cancer survivors and health care providers in Australia to develop a core set of patient-reported outcomes for cancer survivorship research [23]. Our research complements and extends the Australian study by including the views of family/friend caregivers and health system decision-makers, and identifying a broader range of outcomes such as those that may be assessed at the system or population levels.

This study was undertaken with the aim of informing future research, specifically the selection of relevant outcomes, in cancer survivorship. It is important to note that some of the outcomes identified may be easier to assess than others. For example, validated patient-reported instruments exist to assess cancer-related fatigue and fear of cancer recurrence such as the Functional Assessment of Chronic Illness Therapy Fatigue Scale (FACIT-F) [24,25] and Cancer Worry Scale [26], respectively. Other identified outcomes that may be more challenging to measure. For example, measuring coordination of care will depend on the perspective (e.g., patient, provider, or system) and data source (e.g., primary versus secondary data), and likely requires multiple measures to comprehensively assess this construct [27]. Similarly, reintegration after cancer treatment, arguably the stakeholders’ main priority outcome for future research, is a complex construct that would require multiple measures to assess in a holistic and meaningful way. Ore and Foli [28] recently published a concept analysis on reintegration post-cancer treatment that demonstrated the dynamic and evolving nature of this outcome, which involves a reorganization of roles and personal abilities to create a new normal and post-treatment identity. The authors provide some suggested tools to help assess this concept such as symptom scales as well as the Self-Perception and Relationships Tool [29] and the Reintegration to Normal Living Index [30]. The latter is a provider-reported tool used mainly in rehabilitation settings to assess the degree to which individuals who have experienced traumatic or incapacitating illness achieve reintegration into normal activities. Future research is needed to improve the measurement of many of these outcomes including the development and refinement of instruments or indices that attempt to measure these outcomes from primary and secondary data.

Notably, for a number of the outcomes identified in this study, evidence-based interventions already exist to address the relevant need. For example, RCTs have demonstrated that both physical activity and psychological interventions reduce fatigue in cancer survivors [31,32,33]. Similarly, RCTs of psychological interventions, particularly contemporary and blended cognitive behavioral therapies, have shown small but robust effects on fear of cancer recurrence [34,35]. Thus, the challenges in this regard pertain to the implementation of this evidence into cancer clinics and practice, and/or survivors’ abilities to access these services and resources where available. Both challenges are highlighted in the system-level outcomes participants perceived as particularly important: access to care and use of evidence-based practices, supports, and services. Nationally, stakeholders including cancer research funders in Canada have prioritized the need for robust implementation science in cancer survivorship to ensure that evidence-based practices are indeed translated into programs and services [36]. Conversely, there are few evidence-based interventions to improve a number of the outcomes identified. For example, systematic reviews on interventions to improve coordination and continuity of care between oncology and primary care have found that most studies show no changes in any patient, provider, or system outcomes [4,5]. Certainly, continuity of care is a complex system construct that is not necessarily easy to define or measure [37].

While this study sought to inform future research in cancer survivorship in a practical way, the findings also reflect the shift to more person-centered care and have implications around the use of patient-reported outcome measures (PROMS) and patient-reported experience measures (PREMS) in cancer survivorship care. Indeed, all of the outcomes in the psychosocial, physical, economic, and informational domains should be assessed from the patient perspective, though some may be more challenging to assess than others, as noted above. Notably, nine of 10 Canadian provinces have implemented some level of PROM/PREM reporting, using the Edmonton Symptom Assessment System-revised (ESAS-r) [38]. However, important gaps remain including the screening of symptoms across the entire cancer care continuum, not simply during treatment [39]. The findings from this study suggest that monitoring and improving cancer survivors’ experiences and outcomes will require instruments other than ESAS-r, which does not assess any of the economic and informational outcomes important to survivors, nor does it assess cognitive impairment, sexual health, fear of recurrence, or reintegration after cancer treatment. The identification of a minimal set of instruments to assess concerns in the post-treatment period, alongside clear implementation guidance, may be needed to ensure that survivors’ concerns are met in a timely and comprehensive way. Given the challenges associated with implementing ESAS-r in a consistent and standardized way [39], the use of electronic and mobile health platforms may improve the collection of patient-reported data in the future.

This study has a number of limitations. First, asking participants about ideal or preferred outcomes during follow-up care, without a specific intervention in mind, is somewhat of an abstract request. Thus, participants in this study often had to tie this task to their personal or professional needs and experiences to answer in a more concrete sense. Another study design may have provided a broader range of, or different, relevant outcomes. At the same time, we included a wide range of stakeholders (survivors, family/friend caregivers, oncology providers, primary care providers, and cancer system decision-/policy-makers) from across Canada, who had experience with a range of cancer types and subpopulations. Thus, these outcomes represent common needs/concerns experienced across cancer types and subpopulations, and should provide clear direction for researchers in terms of outcomes they should address in future research aimed at improving the survivors’ experiences and outcomes. Second, this study was undertaken in Canada only, and therefore the relevant outcomes may not be representative of jurisdictions whose health care systems differ substantially from Canada. However, patient-level outcomes such as fatigue, cognitive impairment, and fear of recurrence are unlikely to differ substantively across jurisdictions, as evidenced by a recent work in both Australia and the United States [23,40]. The identified system level outcomes such as access to care and coordination may not be as germane elsewhere. Third, only 27.8% of survivors and family/friend caregivers were men, with no male participants in a caregiving role. Thus, the results may not fully reflect men’s perspectives on what is most important in the post-primary treatment phase of care. Despite these limitations, this study addresses an important issue in study design: namely, the identification of outcomes relevant to stakeholders who work in or who experience our health care systems. Prior research has demonstrated that outcomes important to patients and other stakeholders are misaligned with those actually assessed in research studies [12,13,14,15].

## 5. Conclusions

This study identified 13 outcomes that the stakeholders viewed as important to address in future research on post-treatment follow-up care. These outcomes were identified across stakeholder groups and jurisdictions, and should inform future research in this area. This includes both future trials of new and existing interventions as well as the evaluation of programs in cancer survivorship care. Future research should also identify and examine categories of survivors (e.g., based on factors such as cancer type, clinical course, and phase of care) to identify what is most important to survivors in these different categories [41]. This would allow for a more personalized approach to delivering needed services and support. Studying stakeholder-relevant outcomes is critical to reducing research waste and enabling the translation of findings into clinical practice and programs for the growing population of cancer survivors.

## Figures and Tables

**Table 1:** Participant characteristics (*n* = 44).

Characteristic	*n*	%
Role		
Survivor	11	25
Family/friend caregiver	7	15.9
Healthcare provider ^1^	18	40.1
Decision-maker	8	18.2
Region		
Western provinces (BC, AB, SK, MB)	14	31.8
Central provinces (ON, QC)	17	38.6
Atlantic provinces (NB, NS, PE, NL)	13	29.5
Gender of survivor/caregiver (*n* = 18)		
Woman ^2^	13	72.2
Man	5	27.8
Non-binary	0	0
Age of survivor/caregiver (*n* = 18)		
18–39	5	27.8
40–64	10	55.6
65+	3	16.7
Main cancer type for survivor/caregiver (*n* = 18)		
Breast	4	22.2
Colorectal	3	16.7
Genitourinary	3	16.7
Ovarian	2	11.1
Melanoma	1	5.6
Blood	5	27.8
Years since diagnosis for survivor/caregiver (*n* = 18)		
<3 years	6	33.3
>3 years	12	66.7
Years in practice/decision-making for healthcare provider/decision-maker (*n* = 26)		
1–4 years	4	15.4
5–14 years	10	38.5
15+ years	12	46.2

^1^ Health care providers included oncologists (*n* = 4), surgical oncologists (*n* = 3), allied health professionals (*n* = 2), registered nurses (*n* = 2), and primary care (family physicians, nurse practitioners, and general practitioners in oncology; *n* = 7).^2^ All caregivers were female.

**Table 2:** Summary of stakeholder-relevant outcomes.

Domain	Outcome
Psychosocial	Reintegration after cancer treatment
	2. Fear of cancer recurrence
	3. Anxiety
Physical	4. Fatigue
	5. Cognitive impairment
	6. Sexual health
Economic	7. Return to work
	8. Financial burden
Informational	9. Knowing what to expect
	10. Informational continuity
Patterns and quality of care	11. Access to care
	12. Coordination of care
	13. Use of evidence-based practices

## Data Availability

The data presented in this study are available on request from the corresponding author. The data are not publicly available due to privacy and confidentiality considerations.

## Supplementary Materials

The following are available online at https://www.mdpi.com/article/10.3390/curroncol28040277/s1, Supplementary File S1: Interview guide.
