# Supplementary material for: What Matters in Cancer Survivorship Research? A Suite of Stakeholder-Relevant Outcomes
Source: Curr Oncol. 2021 Aug 20;28(4):3188–200. doi: 10.3390/curroncol28040277 (PMC8395501; doi:10.3390/curroncol28040277)
Supplement: Supplementary file 1 [file curroncol-28-00277-s001.zip › curroncol-1313936-supplementary.pdf]

Draft Interview Guide - Survivors and Family/Friend Caregivers

Sample wording for the various stakeholder groups is provided below, however, wording of questions will be adapted for individual stakeholders.

<u>Demographics</u>

If the interviewee is a **survivor**:

- Can you tell me which age category you fall into, (i.e. <40 years, 41-65 years, or >65 years of age)?
- How would you identify your gender identity?
- What type of cancer were you diagnosed with?
- How much time has passed since your diagnosis (i.e. <1 year, 1-3 years, or >3 years)?
- In what province did you receive your treatment, including follow-up care?

If the interviewee is a **family/friend/caregiver**:

- Can you tell me which age category you fall into, (i.e. <40 years, 41-65 years, or >65 years of age)?
- How would you identify your gender identity?

Now, can you tell me a bit about your family/friend whom you cared for. Specifically,

- Can you tell me which age category they fall into, (i.e. <40 years, 41-65 years, or >65 years of age)?
- What is their gender?
- What type of cancer were they diagnosed with?
- How much time has passed since their diagnosis (i.e. <1 year, 1-3 years, or >3 years)?
- In what province did they receive their treatment, including follow-up care?

1. Tell me about your transition/the transition of your family member/friend after treatment to “routine follow-up care”. How did that transition go?
    a. Were you/was your family member/friend discharged from the cancer centre/clinic? If so, how did this happen?
    b. What communication or supports did you/your family member/friend receive during that time?
    c. Who is now responsible for your follow-up care/the follow up care for your family member/friend?

2. From your experience, what were or are the most pressing issues you/your family member/friend faced (or still face) in the time period after cancer treatment?
    a. Physical concerns (e.g., pain, fatigue, sexual function, lymphedema)
    b. Emotional concerns (e.g., worry about recurrence, anxiety, depression)
    c. Practical concerns (e.g., return to work, tight finances)

3. Let’s think “blue sky” about some intervention aimed to help people after they complete their cancer treatment – some tool or program or service that prepares people for this transition or addresses their main concerns after treatment. What might an ideal intervention look like?

From your perspective, whatever the intervention is, what would an ideal intervention *do* for cancer survivors? What would it address? [Put another way, what would you *expect from* an ideal intervention?]

   a. What would an ideal intervention do for survivors' family/friend caregivers?
   b. Their providers?
   c. The health system at large?

4. Is there anything else you would like to share around this topic that we haven't talked about? Or something you would like to expand on?

Draft Interview Guide - Decision-/Policy-Makers

Sample wording for the various stakeholder groups is provided below, however, wording of questions will be adapted for individual stakeholders.

<u>Demographics</u>

- How many years of experience do you have in your current role?
- Can you confirm which province you currently work in?

1. Within your program/institution/organization/jurisdiction, how are survivors transitioned after treatment to “routine follow-up care”? How does that transition usually go?
   a. Are patients discharged from the cancer centre/clinic? If so, how does this happen?
   b. What communication or supports are provided to patients and their family/friend caregivers at that time?
   c. Who do you consider responsible for the follow-up care of survivors once they have been discharged from the cancer center?

2. From your experience, what were or are the most pressing issues survivors face in the time period after cancer treatment?
   a. Physical concerns (e.g., pain, fatigue, sexual function, lymphedema)
   b. Emotional concerns (e.g., worry about recurrence, anxiety, depression)
   c. Practical concerns (e.g., return to work, tight finances)

3. Let’s think “blue sky” about some intervention aimed to help people after they complete their cancer treatment – some tool or program or service that prepares people for this transition or addresses their main concerns after treatment. What might an ideal intervention look like?

   From your perspective, whatever the intervention is, what would an ideal intervention *do* for cancer survivors? What would it address? [Put another way, what would you *expect from* an ideal intervention?]
   a. What would an ideal intervention do for survivors’ family/friend caregivers?
   b. Their providers?
   c. The health system at large?

4. Is there anything else you would like to share around this topic that we haven’t talked about? Or something you would like to expand on?

Draft Interview Guide - Physicians

Sample wording for the various stakeholder groups is provided below, however, wording of questions will be adapted for individual stakeholders.

Demographics<br>• How many years of experience do you have in your current role?<br>• Can you confirm which province you currently practice in?

1. Tell me about the transition of your patients from treatment to "routine follow-up care". How does that transition usually go?
    a. Do you discharge your patients/are your patients discharged from the cancer centre/clinic? If so, how does this happen?
    b. What communication or supports are provided to patients and their family/friend caregivers at that time?
    c. Who do you consider responsible for the follow-up care of survivors once they have been discharged from the cancer center?

2. From your experience, what were or are the most pressing issues survivors face in the time period after cancer treatment?
    a. Physical concerns (e.g., pain, fatigue, sexual function, lymphedema)
    b. Emotional concerns (e.g., worry about recurrence, anxiety, depression)
    c. Practical concerns (e.g., return to work, tight finances)

3. Let's think "blue sky" about some intervention aimed to help people after they complete their cancer treatment – some tool or program or service that prepares people for this transition or addresses their main concerns after treatment. What might an ideal intervention look like?

    From your perspective, whatever the intervention is, what would an ideal intervention *do* for cancer survivors? What would it address? [Put another way, what would you *expect from* an ideal intervention?]
    a. What would an ideal intervention do for survivors' family/friend caregivers?
    b. Their providers?
    c. The health system at large?

4. Is there anything else you would like to share around this topic that we haven't talked about? Or something you would like to expand on?
